# Doctors’ and nurses’ perceptions of a ward-based pharmacist in rural northern Sweden

**DOI:** 10.1007/s11096-017-0488-5

**Published:** 2017-05-25

**Authors:** Maria Sjölander, Maria Gustafsson, Gisselle Gallego

**Affiliations:** 1 Department of Pharmacology and Clinical Neuroscience, Umeå University, 90187 Umeå, Sweden; 2 School of Medicine, The University of Notre Dame Australia, 160 Oxford Street, Darlinghurst, NSW 2010 Australia

**Keywords:** Clinical pharmacy, Doctors perceptions, Nurses perceptions, Rural health care, Sweden

## Abstract

*Background* This project is part of the prospective quasi experimental proof-of-concept investigation of clinical pharmacist intervention study to reduce drug-related problems among people admitted to a ward in a rural hospital in northern Sweden. *Objective* To explore doctors’ and nurses’ perceptions and expectations of having a ward-based pharmacist providing clinical pharmacy services. *Setting* Medical ward in a rural hospital in northern Sweden. *Method* Eighteen face-to-face semi-structured interviews were conducted with a purposive sample of doctors and nurses working on the ward where the clinical pharmacy service was due to be implemented. Semi-structured interviews were digitally recorded, transcribed and analysed using thematic analysis. *Main outcome measure* Perceptions and expectations of nurses and doctors. *Results* Doctors and nurses had limited experience of working with pharmacists. Most had a vague idea of what pharmacists can contribute within a ward setting. Participants, mainly nurses, suggested inventory and drug distribution roles, but few were aware of the pharmacists’ skills and clinical competence. Different views were expressed on whether the new clinical pharmacy service would have an impact on workload. However, most participants took a positive view of having a ward-based pharmacist. *Conclusion* This study provided an opportunity to explore doctors’ and nurses’ expectations of the role of clinical pharmacists before a clinical pharmacy service was implemented. To successfully implement a clinical pharmacy service, roles, clinical competence and responsibilities should be clearly described. Furthermore, it is important to focus on collaborative working relationships between doctors, nurses and pharmacists.

**Electronic supplementary material:**

The online version of this article (doi:10.1007/s11096-017-0488-5) contains supplementary material, which is available to authorized users.

## Impacts on practice

- To successfully implement clinical pharmacy services in hospital wards, roles, clinical competence and responsibilities should be clearly described and specified.
- When implementing clinical pharmacy services in a hospital, it is important to focus on collaborative working relationships between doctors, nurses and pharmacists.
- To be able to integrate clinical pharmacists into health care teams, it is important that other health care professionals understand their required skills and competence, as well as having clearly defined expectations of the service they provide.

## Introduction

The role of pharmacists has experienced significant changes in recent decades. In a hospital setting, pharmacy services have changed from traditional dispensing roles to become patient-centred services [1]. Studies have demonstrated that the provision of clinical pharmacy services (CPSs) can contribute to a significant reduction in adverse drug events (ADEs), [2] length of stay and readmissions to hospital [3, 4]. However, as described by Makowsky et al. [5] even though clinical pharmacists have the potential to improve medication safety in hospital settings, they are still an underutilised resource. While in countries such as the United States, [6] the United Kingdom [7] and Australia [8] ward-based pharmacists are commonplace, until recently very few Swedish clinical pharmacists worked in hospitals providing CPSs. The degree of implementation of CPSs in hospitals in Sweden has varied. In 2009, after positive results from a trial exploring the effectiveness of interventions carried out by ward-based pharmacists in reducing morbidity and the usage of hospital care for elderly patients, CPSs were implemented in some hospitals in the south of Sweden [9].

In Västerbotten County, one of five counties situated in Norrland, the most northerly region of Sweden, CPSs have been provided on a number of wards at two regional hospitals since 2003. In 2014 a request was made for this service to be implemented on the medical ward of a rural hospital in the region. The clinical pharmacy service included medication reconciliation, medication review, and participation in ward rounds. Before the implementation, a study was designed to investigate if medication reviews conducted by a clinical pharmacist as part of a ward team can reduce drug-related problems (DRPs) among people admitted to the medical ward at a rural hospital. However, since this involved a change in current practice, it was important to understand the factors that may have an impact on the implementation of the CPSs [10]. Makowsky et al. [11] described how the integration of pharmacists into core health care teams appears to assist better team decision-making around drug therapy, positive patient outcomes, improving patient safety and improving continuity of care. Research in primary care settings in the United States, [12] Canada [13] and the United Kingdom [14] has highlighted the importance of understanding primary care practitioners’ expectations and perceptions of pharmacy services. This is important in order to develop collaborative relationships. However, to date limited research has been conducted in Sweden to understand the working relationships and perceptions and expectations of doctors and nurses prior to the introduction of a CPS on a hospital ward.

### Aim of the study

This study aimed to explore doctors’ and nurses’ perceptions and expectations of having a ward-based pharmacist providing clinical pharmacy services in a rural hospital in northern Sweden.

### Ethics approval

The study was approved by the Regional Ethical Review Board in Umeå (registration number 2014/322-31Ö). Written consent was obtained from all study participants. All transcripts were de-identified and all data were kept confidential.

## Method

This study adhered to the consolidated criteria for reporting qualitative research (COREQ) [15].

### Setting and context

This study took place on the general medical ward of a hospital in rural northern Sweden. The ward has 18 beds and different categories of patients are admitted to this ward. However, most patients are older and have multiple comorbidities. The region is sparsely populated, with a population density of fewer than five people per square kilometre [16]. It is situated in the middle of the Västerbotten County, which has a catchment area covering 55,186 km^2^ (about the size of Denmark) and is populated by about 259,239 inhabitants. Of these, the majority live in or near the city of Umeå, which has a university hospital. However, around 70,000 people live outside the urban areas. The most distant point in the county is about 300 km from the hospital and due to its geographical location it is also the base for the ambulance helicopter [16].

Before the interviews were conducted on the medical ward, no clinical pharmacist had provided patient care services at this hospital. However, since 2015, prescriptionists (“receptarie” in Swedish) have been in charge of delivering drugs to the hospital once a week from a nearby regional hospital (128 km away). The prescriptionists’ main tasks involved checking inventory, ordering and unpacking drugs and interacting with the nurses about these issues.

### Participant recruitment

Purposive sampling [17] was used to identify doctors and nurses working on the ward where the clinical pharmacy service was due to be implemented. All doctors and nurses on the ward were invited to participate. The clinical nurse manager in charge of the ward scheduled all the interviews. Days and times were chosen to best suit the workload and staffing of the ward.

All interviewees were given information about the study and were informed that participation was voluntary. An interview schedule was developed with a list of topics to be discussed during the interviews. Topics included: the role of pharmacists, prior experience of working with pharmacists and the perceptions and expectations of having a pharmacist on the ward (see Appendix 1). There are no formal requirements to be able to work as a clinical pharmacist in Sweden, both pharmacist (“apotekare”) and prescriptionist (“receptarie”) can work as clinical pharmacist. However pharmacist (“apotekare”) are more likely to have this role. The term used during the interviews was pharmacist (“apotekare”).

### Data collection

Semi-structured interviews were conducted between April and May 2015 at the hospital by the first author (MS). Due to the confidential nature of the information revealed by the respondents, there was an emphasis on reassuring each respondent that anonymity was guaranteed. All participants signed a consent form. Interviews were digitally recorded with the permission of the interviewees, transcribed verbatim and translated into English. Background data (age, gender, years working at the hospital and years in their current role) were also collected on all participants.

### Data analysis

Data collection involved several stages. First, author MS read and reread the transcripts. At this stage, descriptive codes were applied to the data. Subsequently, based on the first order codes, a coding framework was developed to organise the interview data. Codes were compared and discussed by authors MS & GG until a consensus on a coding framework was reached. Coding and consensus meetings were performed iteratively for the first four interviews. At a later stage, the codes were grouped into themes. Through consensus meetings, the most relevant themes were identified.

## Results

In total, 18 people were invited, and all agreed to participate. An equal number of medical doctors and nurses were interviewed. Sixty-seven percent were females and one-third were 50 years or older. Table 1 describes the characteristics of the study participants. Two broad themes were identified: (1) Role specification with two subthemes: (a) traditional roles versus patient-centred care and (b) working relationships; and (2) Expected outcomes with four subthemes: (a) impact on patient care, (b) drug knowledge, (c) workload and (d) “I think this feels just unnecessary”—negative views.

**Table 1:** Participants’ characteristics

Characteristic	n (%)
*Gender*	
Female	12 (66.7)
*Age range*	
20–29	3 (16.7)
30–39	5 (27.8)
40–49	4 (22.2)
50–59	5 (27.8)
60+	1 (5.6)
*Professional background*	
Medical doctor	9 (50.0)
Nurse	9 (50.0)
*Years working at the hospital*	
0–9	5 (27.8)
10–19	6 (33.3)
20–29	3 (16.7)
30 +	4 (22.2)
*Years in current role*	
0–9	9 (50.0)
10–19	5 (27.8)
20–29	3 (16.7)
30+	1 (5.6)

### Role specification

#### Traditional roles vs patient-centred care

Even though the term pharmacist (“apotekare”) was used during the interviews most participants were not able to differentiate between pharmacists (“apotekare”) and prescriptionists (”receptarie”). Participants described the pharmacist role in different ways. Some had limited experience or a vague idea of what pharmacists can or are allowed to do on a ward. Others—mainly nurses—described traditional roles such as inventory and drug distribution, as illustrated by this quote: “… to keep the drugs or the store room for drugs in order and make sure we don’t run out of drugs, that they are ordered on time. Yes. Unpack them when they arrive maybe.” (Nurse 8).

Some participants expect ward-based clinical pharmacists to educate staff and share their “drug knowledge”. One participant noted: “There are lots of medications that we are not very familiar with. Or we have heard of or seen about them at some point, but we know nothing more about the treatment. Perhaps they will be able to help us, someone to ask…” (Doctor 9)

Views on the patient care role of the pharmacist also varied. Some participants described how they did not expect the pharmacist to have direct contact with patients: “I imagine that they might not be very much directly together with the patient.” (Doctor 1) Others questioned whether they can have access to the patients’ medical records: “… access to medical records and so. I don’t know, when it comes to authorisation, what access they have.” (Nurse 1) Conversely, some (mainly doctors) described how the pharmacist may have a role in patient education as evidenced by these quotes:

I hope that there is time and space for that person to also meet with patients and discuss [with them] what they have understood about their medications and how they actually take [them]. (Doctor 7)

They have a greater opportunity to work with the patient to go through their medicines and like make sure that they understand what it’s about and so on. (Doctor 8)

#### Working relationships

Familiarity or previous experience working with a pharmacist demonstrated a better understanding of their role. Some participants (mainly doctors) were able to describe how decisions are made in a “consultative manner” and described very specific tasks: “Look through the patients’ total drug treatment, give advice and views on it and also participate when we want to change, prescribe or discontinue medications, and be a support and give advice in connection with that.” (Doctor 3)

One of the doctors interviewed raised the issue about the clinical relevance of the pharmacists’ recommendations. This was based on the doctors’ experience with pharmacists in an out-patient pharmacy using a web-based interaction program which identifies all drug interactions. This doctor described how he receives phone calls from outpatient pharmacists asking about interactions which he considered had no clinical relevance: “… they [pharmacists] called all the time and asked: ‘Should they really have a beta blocker and an ACE inhibitor? They don’t go together.’ But that is how heart failure should be treated, so… Well it could be like that, that you get stuck in those kinds of things…” (Doctor 1)

However, another doctor who had previously worked with pharmacists in a different hospital mentioned: “… but my experience of pharmacists was that they could give some more information but that it was not categorical, not that they said ‘No you can’t do that’, but more like counselling. My experience was good.” (Doctor 4)

### Expected outcomes

#### Impact on patient care

The majority of participants took a positive view of the implementation of the new CPS. Some mentioned it would be “exciting and interesting” and “nice to meet new people with a completely different focus”. (Doctor 5) Another participant mentioned: “It is fun to get a new professional group in health care. I think we need more competence specifically about medicines.” (Nurse 2)

I don’t think that there will be any obstacles or conflicts, absolutely not. I am sure we will find some good solution and that they will enjoy it here and that there will be some benefits. (Doctor 9)

Some of the participants mentioned that they hope that patient outcomes and quality of care will improve as illustrated by this quote: “I think that the greatest [expectation] is that you can get the right medicines to the right patients. So I think that would be a very good advantage [sic] both for the patient and also for the treatment in general. We might get the right treatment from the start.” (Nurse 7)

#### Drug knowledge

Some of the participants expected that their knowledge about drugs would improve while working with the pharmacist. This is exemplified by a doctor who explained: “[Apart from increased patient safety] I should take the opportunity to learn a little myself too.” (Doctor 6) Some also mentioned that patients’ knowledge could improve if the pharmacist works with them.

[The clinical pharmacist] will probably bring increased knowledge among those of us who participate in the rounds, simply. Not only doctors, but also other groups of staff. (Doctor 3)

However, some doctors (mainly experienced doctors) were concerned that having a pharmacist may mean losing knowledge or junior doctors not gaining competence on drugs. “If this in some way takes the training [to talk to patients about drugs] away from the junior doctors, it will not be good either. Then you will rely on having another category that will manage that.” (Doctor 8)

#### Workload

Participants also voiced different views on how they believe having pharmacists as part of the ward team might impact on their workload. Some of the senior doctors were concerned that ward rounds or hospital discharge might take longer, at least to begin with.

… probably it will mean that the rounds will take a little longer as a result of another aspect that must be considered, additional viewpoints. (Doctor 3)

Junior doctors believed that having a ward pharmacist would not have an impact on the workload. “It might perhaps take longer to do the round if you are going to discuss more, but that’s not really workload. It’s good, you might not have to check things up and might not have to keep on wondering, but you could maybe solve more things.” (Doctor 1)

Many nurses expected a positive effect on their workload since they anticipated that the pharmacist would do some of their tasks (i.e. ordering drugs, checking expiry dates, keeping the store room for drugs in order). One participant mentioned: “[Pharmacists] can hopefully order drugs. That is something that we [nurses] do today… the kind of things that take time for us that we actually don’t have to do. So if it’s possible to shift that to another category, then that’s incredibly good.” (Nurse 1)

#### “I think this feels just unnecessary”: negative views

While most attitudes were positive, one participant expected no positive outcomes at all and felt that this new service was just unnecessary and that the money should be spent on something else. On the question of whether this person thought that a clinical pharmacist could contribute something, the answer was: “No, no. I must say, I think this feels just unnecessary. I don’t know… we are too small a ward for this to be needed. I think that the money should be spent on something else within the county council.” (Nurse 4)

A second nurse mentioned that nurses will be fired and pharmacists employed instead. “It has been discussed before that it should be a pharmacist that should manage the storage room for drugs. As I said then, as long as it’s not a disadvantage; that is, that it’s seen as meaning that we can take away a nurse because we have a pharmacist to do this. I don’t want that.” (Nurse 9)

## Discussion

This study explored doctors’ and nurses’ perceptions and expectations of having a ward-based pharmacist providing CPSs in a rural hospital in northern Sweden. Study results found that unfamiliarity with pharmacists, their clinical skills and unfulfilled expectations about reduced workload might be barriers to a successful implementation of the clinical pharmacy service. On the other hand, participants’ mostly positive attitudes are likely to be a facilitator. To date, studies have explored the perceptions of doctors and nurses using cross-sectional surveys [18–20]. Most qualitative studies have explored the perceptions of physicians in primary care settings. Lauffenburger et al. [21] and McGrath et al. [22] explored the perceptions of primary care practitioners in the USA, and Hughes and McCann explored these perceptions in Ireland [23]. Makowsky explored collaboration between pharmacists, physicians and nurse practitioners on a Canadian hospital ward [11]. However no qualitative studies have been carried out in hospital settings in Sweden. This study provided the opportunity to explore doctors’ and nurses’ perceptions and expectations of having a ward-based clinical pharmacist before the service was implemented.

The results showed that participants were uncertain about the ward-based clinical pharmacist role and unclear about their clinical skills and competencies. As practitioners begin working together, each may have role expectations about the other that are based on past experiences, stereotypes, and educational backgrounds [24]. For CPSs to be successfully implemented on this ward it is important to raise professional awareness and recognition [25]. Besides role specification [26], trustworthiness [27] is a factor considered to affect the extent to which health professionals collaborate and are willing to delegate and share responsibilities. Indeed, uncertainty about the clinical pharmacists’ competence, and concerns about their ability to distinguish between clinically relevant or irrelevant questions, were described in the present study. If useful recommendations are made consistently over time and the pharmacist is able to demonstrate their competence, physicians’ trust may develop. Gaining trust is important, but can only be gained from high-quality clinical recommendations that improve patient outcomes [28]. Hence this highlights the importance of clinical pharmacists having appropriate training and clinical experience in direct patient care. In Sweden, there are no formal requirements for a pharmacist to work as a ward-based clinical pharmacist except for a Bachelor or Master of Science degree in Pharmacy. There are no residency training programmes for pharmacy graduates who want to work in the hospital sector. However, to ensure that pharmacists are prepared, trained and have the necessary experience in direct patient care, enhanced training in clinical pharmacy is desirable, and postgraduate education is offered in Sweden. A standardised set of pharmaceutical services is being offered to patients and prescribers (see Table 2), as the majority of Swedish clinical pharmacists work in a similar way to one another [29].

**Table 2:** Clinical pharmacy services provided in the ward

When	How often	Activity
At admission	Once for each patient	Admission medication reconciliation
During hospital stay	As long as the patient is still in the hospital ward	Medication review and monitoring
	Depending on need and time	Patient counselling
	When clinical relevant DRPs are found	Discussion with responsible physician in ward rounds

*DRP* drug related problem

Doctors expressed worries about losing competence regarding knowledge of medicines. This is consistent with previous research concluding that being threatened and fears of dilution of professional identities are barriers that could also hinder collaborative working relationships (CWRs) [22, 30]. Further, one nurse voiced her concern about losing her job and being replaced. The CWR conceptual model described by McDonough [25] describes how individual, context and exchange characteristics influence the level of collaboration among health care professionals. This study allowed us to understand the “context” which appears to see the pharmacist role in distribution, drug-focused (information about medicines, side effects) and not towards patient-centred roles (patient education). Despite the reservations and barriers previously described, participants also conveyed positive attitudes, and thought it would be “exciting” and “interesting” to work together with clinical pharmacists. This is encouraging, as old habits and difficulties adapting to new things have been mentioned as barriers in earlier studies [30].

Some of the relevant factors identified in this study are potentially modifiable and have informed the introduction of the CPSs in this hospital ward. For instance, since the lack of understanding of the pharmacist role was a potential barrier, a talk was given to the ward staff (nurses and doctors) explaining in detail what the CPS encompassed.

### Strengths and limitations of the study

One of the limitations of this study is that things may have been “lost in translation” as the interviews were conducted in Swedish and then translated into English. There may also be cultural nuances that are not captured by doing the data analysis in English. Doctors and nurses who participated in the study may not be the same as the ones that will be around once the CPSs are rolled out due to staff turnover.

A strength of this study was the participation of both doctors and nurses from the ward. Hence all viewpoints were explored. It was also a unique opportunity to find a hospital where no pharmacy services are available and drug supply is provided by a regional hospital. It is important to note that while the hospital has not had CPSs implemented, two of the physicians have had contact with clinical pharmacists before.

## Conclusion

The results showed that the participants’ expectations of the clinical pharmacist role were unclear. Unfamiliarity with pharmacists, their clinical skills and unfulfilled expectations about reduced workload might be a barrier to a successful implementation of the CPS. On the other hand, participants’ mostly positive attitudes are likely to be a facilitator.

## Electronic supplementary material

Below is the link to the electronic supplementary material.

Supplementary material 1 (PDF 267 kb)
