# Supplementary material for: Doctors’ and nurses’ perceptions of a ward-based pharmacist in rural northern Sweden
Source: Int J Clin Pharm. 2017 May 25;39(4):953–9. doi: 10.1007/s11096-017-0488-5 (PMC5541103; doi:10.1007/s11096-017-0488-5)
Supplement: Supplementary file 1 — Supplementary material 1 (PDF 267 kb) [file 11096_2017_488_MOESM1_ESM.pdf]

Electronic supplementary material – Appendix 1

Title: Doctors' and nurses perceptions of ward-based pharmacist in rural Northern Sweden

Journal: International Journal of Clinical Pharmacy

Authors

Maria Sjölander BScPharm, MPH, PhD, Lecturer

Maria Gustafsson MScPharm, PhD, Lecturer

Gisselle Gallego BPharm, PhD, Senior Research Fellow, Visiting Professor

Corresponding Author:

Maria Sjölander, Department of Pharmacology and Clinical Neuroscience, Umeå University, Umeå, Sweden, SE-901 87 Umeå, Sweden. Phone: +46 90 785 39 04; Fax: +46 90 12 04 30

E-mail: maria.sjolander@umu.se

Interview schedule

Background questions

1. What is your education?
2. What is your position here?
3. How long have you been working at this hospital?
4. How long have you been working as… (from question 2)?
5. In which wards do you work? (If applicable)
6. Sex (interviewer to note)
7. Age (provide brackets) 20-29, 30-39, 40-49, 50-59, 60+

A. Role description

1. I'd like to start with a broader question. What are your experiences of pharmacists in general?

How would you, with your experience, describe what a pharmacist does?

Prompt – ask them to use their own examples (based on experience) if necessary.

Ask about pharmacists working in community pharmacy**.**

Have you had contact with pharmacists in your work? What was the purpose?

B. Personal experience of working with a pharmacist (Ask if they mentioned they had worked with pharmacists in the past)

2. What experiences do you have from working with pharmacists?

    If experience: How did you experience working with a pharmacist?

3. In what way did the pharmacist contribute to the work?

C. Expectations

4. What are your expectations of having a pharmacist on the ward?

Prompt (what do you think they will be doing on the hospital ward, what do you think a pharmacist could contribute with and how may this impact on your workload – if applicable).

If a manager, ask about the non-clinical outcomes.
